# Enhancing the Thermostability of Engineered Laccases in Aqueous Betaine-Based Natural Deep Eutectic Solvents

**DOI:** 10.1021/acssuschemeng.1c07104

**Published:** 2021-12-29

**Authors:** Simona Varriale, Astrid E. Delorme, Jean-Michel Andanson, Julien Devemy, Patrice Malfreyt, Vincent Verney, Cinzia Pezzella

**Affiliations:** † Biopox srl, Viale Maria Bakunin 12, Naples 80125, Italy; ‡ CNRS, SIGMA Clermont, ICCF, Université Clermont Auvergne, F-63000 Clermont-Ferrand, France; § Department of Agricultural Sciences, University of Naples “Federico II”, Via Università, 100 Portici 80055, Italy

**Keywords:** Laccase, Deep eutectic solvent, Enzyme stability, Molecular docking, Betaine, Polyols

## Abstract

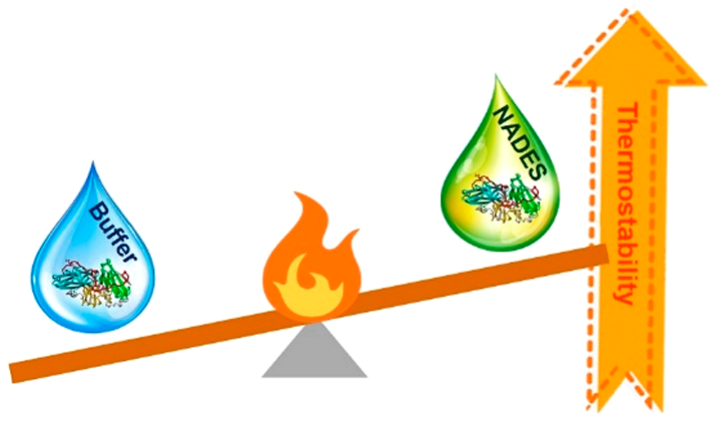

In recent years, natural deep eutectic solvents (NADESs) have gained increasing attention as promising nontoxic solvents for biotechnological applications, due to their compatibility with enzymes and ability to enhance their activity. Betaine-based NADESs at a concentration of 25 wt % in a buffered aqueous solution were used as media to inhibit thermal inactivation of POXA1b laccase and its five variants when incubated at 70 and 90 °C. All the tested laccases showed higher residual activity when incubated in NADES solutions, with a further enhancement achieved also for the most thermostable variant. Furthermore, the residual activity of laccases in the presence of NADESs showed a clear advantage over the use of NADESs’ individual components. Molecular docking simulations were performed to understand the role of NADESs in the stabilization of laccases toward thermal inactivation, evaluating the interaction between each enzyme and NADESs’ individual components. A correlation within the binding energies between laccases and NADES components and the stabilization of the enzymes was demonstrated. These findings establish the possibility of preincubating enzymes in NADESs as a facile and cost-effective solution to inhibit thermal inactivation of enzymes when exposed to high temperatures. This computer-aided approach can assist the tailoring of NADES composition for every enzyme of interest.

## Abstract 2

Aqueous betaine-based NADESs were used to inhibit thermal inactivation of laccases and the stabilizing interactions highlighted by molecular docking.

## Introduction

Laccases are widespread multicopper oxidases catalyzing the oxidation of a broad range of phenolic and nonphenolic substrates with the concomitant reduction of oxygen to water, the only reaction byproduct.^1^ The radical nature of the oxidation gives rise to reactive radical species as primary oxidation products that can evolve through both degradative and synthetic processes.^2^ This feature, together with the large substrate promiscuity of laccases, translated into multifaceted examples of biotechnological applications.^2,3^

One of the challenges in the implementation of laccases in industrial processes is their ability to remain active for a longer time or survive harsh operative conditions, thus resulting in the need of reengineer enzymes to fine-tune their properties toward the end application, hence boosting both productive efficiency and enzyme performance.^4,5^ Recently, flexible surface loops have been identified as potential targets to improve enzyme thermostability by applying modifications aimed at their stiffening.^6,7^ An additional and complementing approach to boost enzyme thermostability consists of exploring microbial diversity by culture mining or metagenomic approaches^8^ searching for extremozymes. On the other hand, the immobilization of enzymes onto solid supports has become a key enabling technology to implement *postsynthesis* their exploitation in industrial processes, promoting enzyme reusability and recovery,^9,10^ while enhancing their thermostability.^11^

Deep eutectic solvents (DESs) are a mixture of a hydrogen bond acceptor (HBA), such as an ammonium salt, and a hydrogen bond donor (HBD), such as polyols and sugars,^12^ resulting in a significantly lower melting temperature compared to one of its individual components. A subset of DESs, termed NADESs, are prepared solely using raw materials of natural origin (such as sugars and amino acids).^13^ The applications of DESs as nontoxic solvents have been increased due to their attractive properties including low flammability, low volatility, facile preparation, high solvability, and compatibility with enzymes.^14,15^ The latter resulted in higher product conversion and enhanced enantioselectivity in a broad range of biocatalytic reactions.^15^ More interestingly, the applications of DESs as cosolvents can also prevent enzyme inactivation typically observed in organic solvents.^12,16^ These hints led to the possibility to incubate enzymes in DESs as an alternative *postsynthesis* approach to avoid their thermal inactivation at high temperatures.^17−19^

To date, only a few works on the use of DESs as alternative solvents for laccase stabilization have been reported.^20−22^ Delorme et al.^20^ have demonstrated that by tailoring the NADESs based media the thermostability of *T. versicolor* laccase could be significantly enhanced. Analogously, by applying a molecular docking approach, Toledo et al.^22^ have found a correlation between the interaction of NADES components in the active site and the observed increase in laccase activity.

A thorough comprehension of the effects of DES composition on enzyme stability and activity would be helpful to tailor these green solvents to specific biocatalytic processes. A computer-aided approach based on docking simulation was tested in this work to gain more insights into this structure–function relationship. POXA1b laccase from the white-rot fungus *Pleurotus ostreatus* was chosen as the target enzyme. It is a high redox potential laccase (HRPL) widely applied in different fields^23−25^ due to its industrial-suited peculiarities, such as the stability and activity in a wide range of pHs (3–9) and temperatures (25–65 °C) and high production level in heterologous hosts.^26^ More importantly, a collection of evolved variants displaying improved phenotypes has been developed,^27^ and five of them were tested in this work to investigate the NADES effect.

Our findings will contribute not only to further widen the applicability window of POXA1b laccase but also to assess the effect that few mutations on a common enzyme scaffold exert on the interaction with tested NADES.

## Materials and Methods

### Materials

2,2′-Azino-bis(3-ethylbenzathiazoline-6-sulfonic) acid (ABTS) was purchased from AppliChem GmbH (Germany) and was used as the substrate to measure laccase activity. For the evaluation of laccase activity in NADES media, the enzymes were incubated in five different aqueous NADES media with a 2HBA:1HBD molar ratio. Betaine (Bet) (Alfa Aesar, Thermo Fisher GmbH, Germany) was used as HBA for each NADES media. Sorbitol (Sor) (neoFroxx GmbH, Germany), xylitol (Xyl) (Carbosynth Ltd., United Kingdom), glycerol (Gly) (Biochem Chemopharma, France), ethylene glycol (EtG) (VWR International, France), and meso-erythritol (Ery) (Molekula GMBH, Germany) were used as HBD. NADESs were prepared as aqueous solutions of 50 wt % NADES and 50 mM phosphate buffer (pH 7) as previously described by Delorme et al.^20^ A 1 mL laccase incubation solution was then prepared with 500 μL of the aqueous 50 wt % NADES solutions and 500 μL of a 50 mM phosphate buffer (pH 7) solution containing 2.5 g L^–1^ of laccase. The final concentrations of NADES and laccase in the incubation solution were 25 wt % and 1.25 g L^–1^, respectively. Betaine and sorbitol aqueous solutions were also prepared individually in 15 and 10 wt %, respectively, which corresponded to the same number of moles and molar concentration as in the 25 wt % 2Bet:1Sor solution.

### Laccases Production

Laccases were recombinantly expressed in *Pichia pastoris* and produced through 5 L pulsed fed-batch fermentations.^26^ The cells were removed by centrifuging for 20 min at 7000 rpm at 4 °C, and the supernatant was recovered, concentrated, and dialyzed toward 50 mM Tris-HCl buffer, pH 8, in a Pall multicassette system (10 kDa cutoff membrane) (Pall Corporation, USA). Laccases were stored as lyophilized pellets at −80 °C.

### Laccase Activity in Betaine-Based NADES

Laccases were incubated in a 1 mL phosphate buffer (50 mM, pH 7) aqueous solution with betaine-based NADESs solution at a concentration of 25 wt % at room temperature for 10 min prior to performing laccase activity assays, as reported by Delorme et al.^20^ Laccase activity was assayed using 2,2′-azino-bis(3-ethylbenzothiazoline- 6-sulfonic acid) diammonium salt (ABTS) as the substrate.^28^ The enzymatic reaction was carried out at room temperature by adding 1 μL of the laccase incubation solution in 900 μL of 100 mM citrate buffer, pH 3, containing 2 mM ABTS. The oxidation of ABTS (extinction coefficient: 36,000 mM^–1^ cm^–1^) was monitored at 420 nm using a UV-1600PC spectrophotometer (VWR, Belgium). One unit (U) of laccase activity is defined as the amount of enzyme able to oxidize 1 μmol of ABTS per minute. Results are the mean from three experiments of three different incubation solutions.

### Thermostability of Laccases in Betaine-Based NADES

Laccase thermostability was evaluated in a phosphate buffer (50 mM, pH 7) aqueous solution containing betaine-based NADES at concentrations of 25 wt % with a laccase concentration of 1.25 g L^–1^. The solution was heated at 70 or 90 °C for different intervals of times. For the thermostability tests at 70 °C, aliquots for the assays were taken after 5, 10, 20, 30, and 60 min, and 90 °C, aliquots were taken after 1, 2, 3, 4, and 5 min. Results are the mean of three different thermostability tests.

### Association of HBD-HBA in Aqueous Phase

Sorbitol and betaine molecules were modeled using the OPLS force field^29^ and water molecules with the SPC/E model.^30^ The simulation box is formed by one sorbitol, one betaine, and 2000 water molecules. The potential of the mean force or Gibbs free energy profile was calculated using the extended version (eABF)^31,32^ of the adaptative biasing force (ABF) method.^33,34^ The equilibration period consisted of 100 ps, and the averages of the potential mean force (PMF) curves were performed over 10 ns. The separation intermolecular distance ranged from 3.5 to 12 Å. The standard deviations are estimated to be in the range of 0.8 kcal mol^–1^.

### Computational Analyses of Laccase-HBD Interactions

Small molecule docking (SMD) and protein visualization were performed using the YASARA Structure.^35^ The model of POXA1b was used to build the three-dimensional structures of laccase variants by swapping the residues involved in the mutations. Receptors were then cleaned, and their structures were energy minimized using AMBER14 force field. The ligand three-dimensional structures (betaine, sorbitol, xylitol, glycerol, ethylene glycol, and erythritol) were built from their SMILES strings and cleaned, and their geometry was optimized by the YASARA Structure. SMD was applied on all the laccases by Autodock VINA performing 25 docking runs per simulation which are clustered into distinct conformations, differing by at least 5.0 Å heavy atom RMSD after superposing on the receptor. The best binding model was evaluated in terms of binding energy (more positive energies indicate stronger binding, and negative energies mean no binding) and dissociation constant, both expressed as kcal mol^–1^.^36^

A FoldX plugin for YASARA was used to calculate the changes in the Gibbs free energy (ΔΔ*G*) between POXA1b and laccase variant structures (ΔΔ*G* = Δ*G*_variant_ – Δ*G*_wt_) by applying a force field algorithm based on empirical free energy terms.^37^ The three-dimensional structure of POXA1b and its variant models were energy minimized, optimizing the amino acid side chains to get a lower free energy of the protein by removing van der Waals clashes and negative contacts. The free energy of unfolding of POXA1b (Δ*G*_wt_, kcal mol^–1^) and that of each variant (Δ*G*_variant_, kcal mol^–1^) were calculated for three independent runs. The temperature and ionic strength were set to 298 K and 0.05 M, respectively. The more negative is ΔΔ*G*, the more stabilizing the mutations are. The error margin of FoldX is approximately 0.5 kcal mol^–1^, indicating that ΔΔ*G* values falling in that range are not significant.^38^

### Statistical Analysis

Pearson correlation coefficients (*r*) to measure the strength of the linear relationship between residual activity of laccase incubated in NADES at 70 °C and binding energy spread of the best laccase–HBD complex conformation were calculated using the Statistical Package for the Social Sciences (SPSS19, SPSS Inc., USA) software (POXA1b, *r* = 0.947; EV1, *r* = 0.847; EV2, *r* = 0.936; EV3, *r* = 0.791; EV4, *r* = 0.660; and EV5, *r* = 0.965).

## Results and Discussion

### Effect of HBD on Laccase Activity

On the basis of recent results obtained with *T. versicolor* laccase,^20^ five betaine-based NADES media were chosen and tested at 25 wt % aqueous dilution for their thermostabilizing effect on *P. ostreatus* POXA1b laccase and its five mutants (Table 1), in order to exploit the whole applicative potential of this set of variants in a wide range of fields.

**Table 1:** POXA1b Laccase Variants Used in This Study with Their Improved Properties Highlighted

Laccase variants	Substitution	Property	ref
EV1	V162H, F331Y, A336N	More polar binding site for anchoring negatively charged substrates	Giacobelli et al.^39^
EV2	V162S, F331Y, A336N	More polar binding site for anchoring negatively charged substrates	Giacobelli et al.^39^
EV3	K37Q, K51N, L112F, V148L, P494T	Increased redox potential	Macellaro et al.^40^
EV4	K37Q, K51N, L112F, S285N	Increased stability in wide range of pHs	Piscitelli et al.^27^
EV5	L112F, P494T	Increased thermostability	Miele et al.^41^

NADES are based on betaine as the HBA and sorbitol, xylitol, glycerol, ethylene glycol, or erythritol as HBD, in the ratio 2HBA:1HBD.^20^

POXA1b and its variants were incubated in NADES-based media at 25 °C for 10 min, and the relative activity was compared with that in the reference solution (50 mM phosphate buffer, pH 7) (SI Figure S1). All the enzymes kept nearly the same activity as in the reference solution with a maximum enhancement of 20%. These results confirm the ability of betaine to preserve proteins against inactivation and aggregation^42^ and to improve laccase activity and stability in betaine-based NADES.^20−22^

### Effect of HBD on Laccase Thermostability at 70 °C

**Figure 1:**
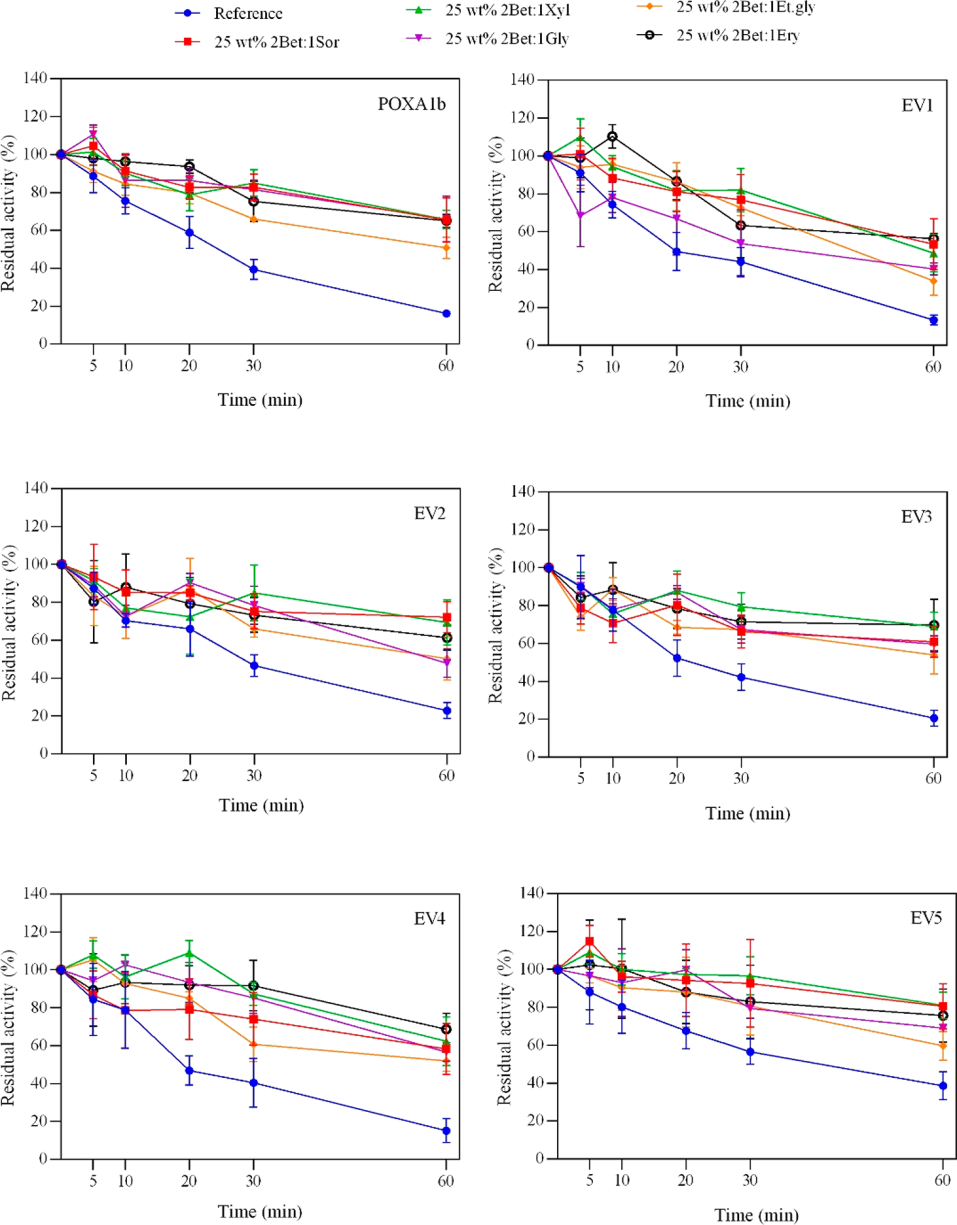
Comparison of thermostability of 1.25 g L^–1^ laccases in 50 mM phosphate buffer, pH 7 aqueous solution in the absence (blue dots), and in the presence of 25 wt % of 2Bet:1Sor (red squares), 2Bet:1Xyl (green triangles), 2Bet:1Gly (purple triangles), 2Bet:1Et.gly (orange dots), and 2Bet:1Ery (black circles) at 70 °C. The residual activity (%) is determined by comparing the activity measured after a set amount of time with the free-NADES phosphate buffer, pH 7.0 and 50 mM at 25 °C (reference, 100%).

When incubated in NADESs at 70 °C, the thermostability of all the tested laccases shows significant improvement (Figure 1). All the applied NADESs had a beneficial effect, although each HBD had different impact on the enzymes’ residual activity. In most cases, laccases showed higher thermostability in the 25 wt % 2Bet:1Sor NADES solution. The presence of NADES quadrupled the residual activity of POXA1b (65% vs 16%) after 60 min of incubation at 70 °C in the 25 wt % 2Bet:1Sor NADES solution. Similar beneficial effects due to incubation in the same NADES solution were observed for EV1 and EV5, while the 25 wt % 2Bet:1Xyl NADES solution was the most advantageous NADES media for EV3 and EV4. This is in accordance with Delorme et al.,^20^ which also described an increase in *T. versicolor* laccase residual activity of nearly 40% and 50% when incubated in 25 wt % 1Bet:3Sor and 25 wt % 2Bet:1Xyl NADES solutions, respectively. These findings demonstrates that the NADES incubation is effective on various laccases, including ones already endowed with high thermal stability, such as EV5.

### Association of HDB and HBA in Aqueous Phase

**Figure 2:**
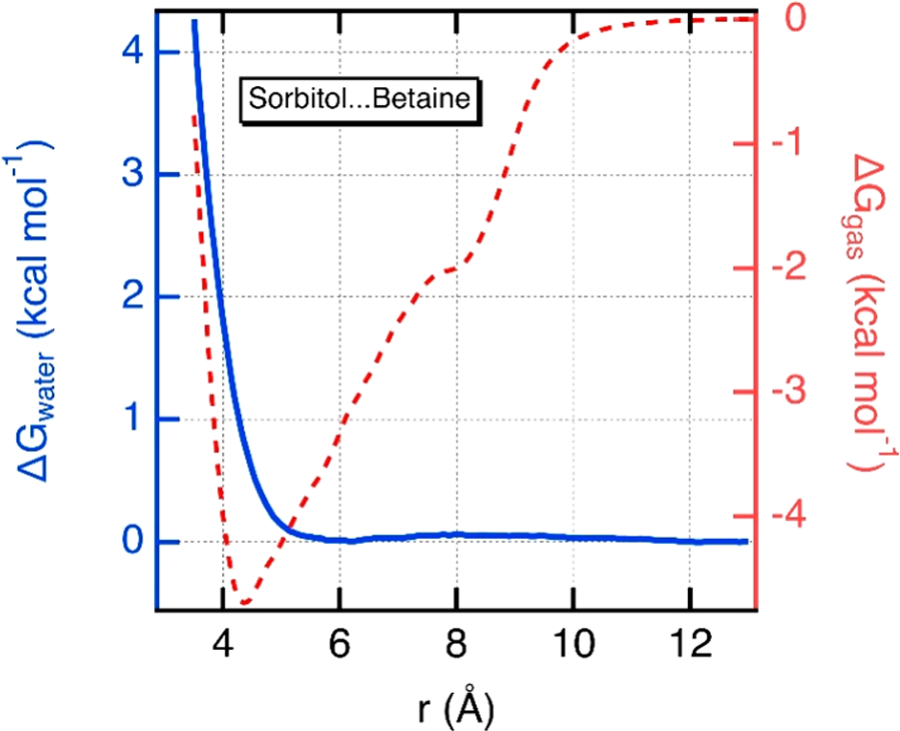
Gibbs free energy profile of the interaction between sorbitol and betaine molecules in water (left axis) and in vacuum (right axis) at 298 K as a function of the intermolecular distance between the centers of mass of both molecules.

Before studying the interaction of the NADES components with laccase, the association between HBD and HBA in water was analyzed in order to know if individual components or clusters of NADES are present in the liquid phase. Figure 2 shows the potential of mean force (PMF) between sorbitol and betaine molecules in water and in vacuum as a function of the separation distance between the center of mass. In vacuum, the PMF curve shows a free Gibbs energy minimum of about −4.7 kcal mol^–1^ at a separation distance of 4.4 Å. This means that the net interaction between these two molecules is favorable leading to an association in vacuum with favorable van der Waals and electrostatic interactions. Interestingly, the same PMF curve calculated in water does not show any free energy minimum indicating that the association between these two molecules is no longer favored in water.

From a thermodynamic viewpoint (SI Figure S2), our results show that the possible formation of the complex (sorbitol–betaine) in water is prevented by unfavorable contributions due to hydration processes. Indeed, the hydration of the complex would be less favorable than hydrating the two species sorbitol and betaine separately.

Other polyols considered in this study have similar structures and would induce similar interactions with betaine and water. Therefore, HBA and HBD are existing as isolated molecules in water, and for the study of the interaction between NADES and laccase, only individual interactions between a single component of NADES and enzyme will be evaluated.

### Computational Analyses of Laccase–HBD Interactions

**Figure 3:**
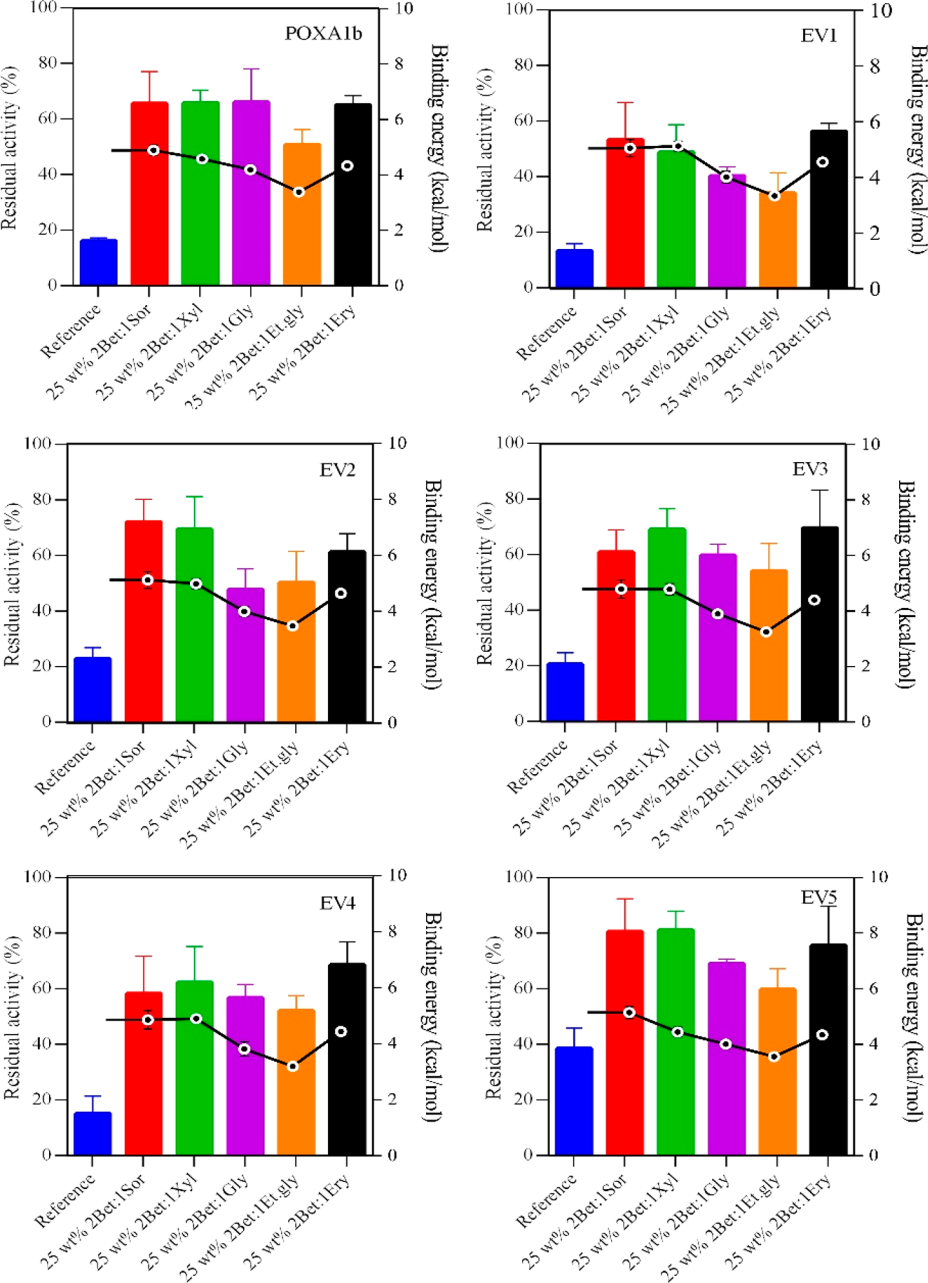
Comparison of thermostability of 1.25 g L^–1^ laccases in 50 mM phosphate buffer, pH 7 aqueous solution in the absence and in the presence of 25 wt % betaine-based NADES obtained using five different HBDs after 60 min at 70 °C (histograms), and the binding energy (kcal mol^–1^) between laccases and polyol molecule of NADES calculated using the YASARA structure (dots). The residual activity (%) is determined by comparing the activity measured after 60 min with the free-NADES phosphate buffer, pH 7.0 and 50 mM at 25 °C (reference, 100%).

With the aim to understand the role of NADES components in stabilization of laccases toward thermal degradation, molecular docking calculations were performed. The distinguishing substitutions of each POXA1b variant were introduced in the POXA1b protein model,^39^ and the respective energies were minimized through YASARA software. The binding energy spread (kcal mol^–1^) of the best laccase–HBD complex conformation was calculated for every couple of laccase variants and each HBD and compared with the results from the residual activity measurements at 70 °C (Figure 3). For all the tested enzymes, a correlation between the residual activities and the binding energy was found (correlation coefficients in the range of 0.660–0.965); the more positive is the binding energy, the higher is the residual activity. In particular, sorbitol, xylitol, and erythritol contribute in a similar extent to the binding energy, which is slightly higher for sorbitol. Conversely, ethylene glycol displays the worse stabilizing effect in terms of binding energy. This behavior is shared among all the tested enzymes. The binding energy for each laccase–betaine complex was also calculated; however, no significant differences were observed among the enzymes, with values assessing at 3.32 ± 0.12 kcal mol^–1^.

**Figure 4:**
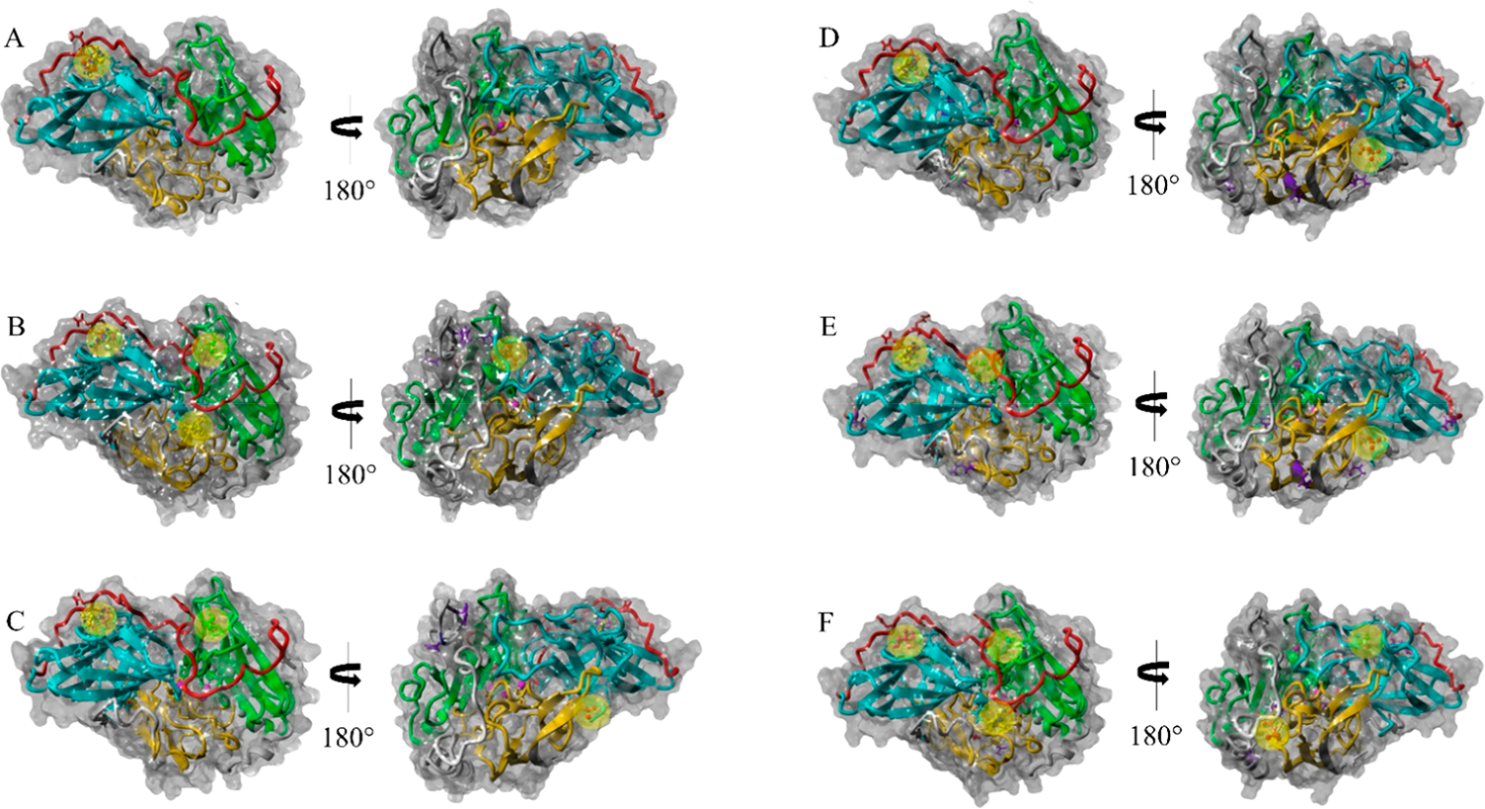
Docking poses of the highest binding energies between laccases and NADES components: (A) POXA1b, (B) EV1, (C) EV2, (D) EV3, (E) EV4, and (F) EV5. Regions of interactions between laccases and NADES components are highlighted in yellow. POXA1b and its variants’ three-dimensional structures can be divided in three domains: domain 1 (amino acid residues 11–130; yellow), domain 2 (amino acid residues 142–283; cyan), and domain 3 (amino acid residues 346–469; green), and the L1 loop (amino acid residues 284–320; red) connecting domains 2 and 3. Mutated residues of POXA1b variants are highlighted in violet.

The localization of NADES components on laccase protein models is displayed in Figure 4, and the details of amino acidic residues involved in the interactions are reported in SI Table S1. In wild type POXA1b, betaine, sorbitol, xylitol, and erythritol interact in the same region of the L1 loop, connecting domains 2 and 3, while ethylene glycol localizes within the active site, possibly explaining the less stabilizing effect due to this molecule. The interaction with glycerol instead occurs in a different region located on the protein surface.

In EV1, a different picture of interactions occurs. Betaine seems to interact in the active site and in particular with H162, the mutated residue of this variant. Xylitol, glycerol, and erythritol interact with the same residues on the exposed region of the L1 loop, while ethylene glycol is predicted to interact with an inner portion of the L1 loop, thus explaining its scarce stabilizing effect on EV1. Interestingly, the residues involved in the interaction with sorbitol, responsible for the highest binding energy observed for this HBD, could identify potential targets for stabilizing interactions.

EV2, EV3, and EV4 display similar scenarios of interactions. As a matter of fact, betaine localizes on protein surfaces, while the interactions with sorbitol, xylitol, glycerol, and erythritol occur on the L1 loop, similarly to EV1 and POXA1b.

The laccase variant EV5 that most benefits from the NADES effect also exhibits a peculiar pattern of interactions. In particular, betaine seems to localize in the C-terminal region, very near to the P494T mutation previously shown to be responsible for the lower protein flexibility of this variant and thus of its intrinsic thermostability.^43^ Interestingly, xylitol and sorbitol that cause the best stabilizing effect interact in the L1 loop, although in a different and peculiar region with respect to the other observed interactions in this loop. This could indicate that although the L1 loop represents a common target for NADES interactions, their stabilizing effect is strictly dependent on the conformational changes of each variant. It can be envisaged that the highest stabilization induced by NADES on the EV5 variant results from the synergic interactions established by each HDB as well as the localization of betaine in a distinguishing interaction site. Furthermore, for the EV5 variant, it is evident that the stabilizing effect increases with the number of hydroxyl groups of the polyol HBD, as observed for *T. versicolor* laccase in ChDHP^22^ and betaine-based DES.^20^

Different kind of interactions, i.e., hydrogen bonds, hydrophobic, cation−π, and ionic interactions have been identified in the best binding models for all the molecule–enzyme couples (S1 Table S1). As a rule of thumb, there is no correlation between the number of interactions and the observed stabilizing effect; on the contrary, it seems that it is the precise combinations of interactions and molecule orientation that are crucial. In fact, although some L1-located residues recur often in the enzyme–molecule interactions, they translate into a different stabilizing effect due to their specific orientation toward the NADES component. Furthermore, besides H162 in EV1, neither of the mutated residues distinguishing each variant is itself involved in the interactions, indicating that these mutations may affect indirectly the conformation of the protein and consequently of the L1 loop.

Taken together, our results highlight the importance of stabilizing interactions on enzyme surfaces and especially in flexible loops to improve enzyme thermostability. By applying a molecular docking approach, Toledo et al.^22^ have found a correlation between the interactions of NADES components in the active site and the observed increase in the enzymatic activity, hypothesizing that such interactions lead to conformational rearrangements of the enzyme that helps the access of the substrate to the enzyme active site.^22^ Both findings are not in contrast if considering that in our case no significant activation of laccases has been found in the presence of NADES, while a notable thermoprotection of enzymes has been observed.

A common trend toward the use of laccases in industry is the design of evolved enzymes able to withstand higher temperatures.^8^ The identification of molecular determinants involved in enzyme thermostability is a still unsolved challenge in protein engineering. Attempts to obtain more robust catalysts have focused on enzyme mutagenesis based on swapping cupredoxin domains, chimeragenesis, or SCHEMA-structure recombination *in vivo*.^7,44^ Recently, flexible surface loops have been identified as potential targets for thermal inactivation, and thus, their modification, aimed at their stiffening, has turned out as an approach to improve enzyme thermostability.^6^ Interestingly, the long L1 loop, connecting domains 2 and 3, is evolutionary conserved in fungal laccases^45^ as well in *P. ostreatus* POXA1b (SI Figure S2). Computational-assisted L1 loop engineering has been recently applied to lcc2 from *T. versicolor* to improve its activity in aqueous solutions and ionic liquids. An increased number of hydrogen bonds, within the loop and between domains 2 and 3, by reducing the flexibility of the loop, has been found responsible for the improved stability of the selected variants.^45^ Analogously, the gain in both hydrogen, ionic, and hydrophobic interactions between the L1 loop residues and NADES components, revealed by our analyses, may be responsible for the observed improvement in enzyme thermostability, highlighting the effectiveness of NADES incubation as an easy and cost-effective *postsynthesis* approach to preserve protein stability.

### Effect of 25 wt % 2Ber:1Sor NADES Components on Laccases Thermostability at 70 °C

**Figure 5:**
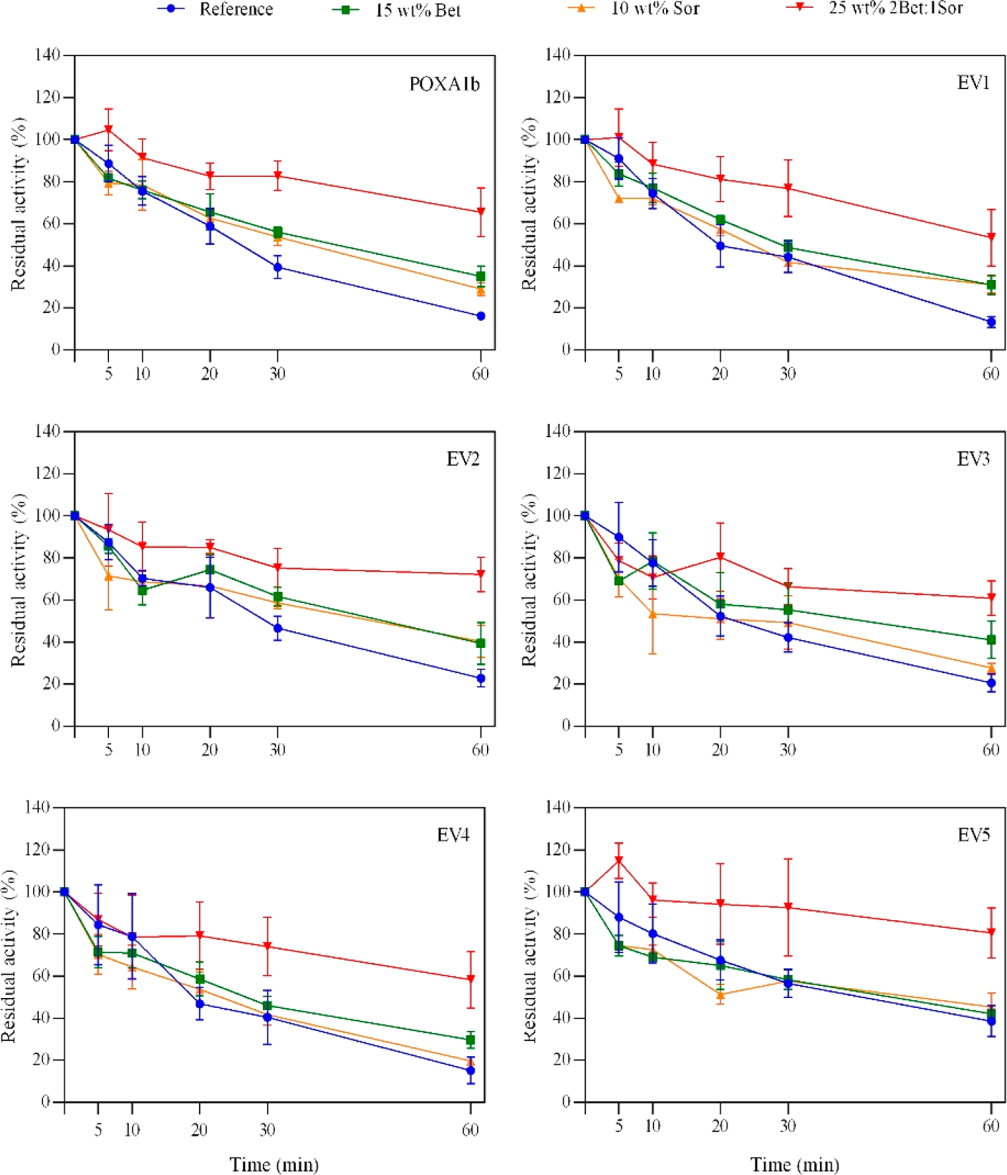
Comparison of thermostability of 1.25 g L^–1^ laccases in 50 mM phosphate buffer, pH 7 aqueous solution in the absence (blue dots) and in the presence of 15 wt % Bet (green squares), 10 wt % Sor (orange triangles), and 25 wt % of 2Bet:1Sor (red triangles), at 70 °C. The residual activity (%) is determined by comparing the activity measured after a set amount of time with the free-DES phosphate buffer, pH 7.0 and 50 mM at 25 °C (reference, 100%).

With the aim of assessing whether the enhancement of laccase stability to high temperature is due to the individual components of NADES or to the combined effect of HBD and HBA, betaine and sorbitol were prepared individually in the same concentration as in the 25 wt % 2Bet:1Sor solution. Laccases were incubated in these aqueous solutions at 70 °C, and their thermal stabilities were assessed (Figure 5). The incubation in 15 wt % betaine increased the thermostability of laccases, while the incubation in 10 wt % sorbitol led to little or no improvement. Conversely, the highest gain in laccases thermostability was achieved when the enzymes were incubated in the 25 wt % 2Bet:1Sor NADES solution. In particular, the EV5 variant retained 80% of its activity when incubated in the NADES solution at 70 °C for 60 min versus a 40% residual activity in the presence of both single components, thus confirming the combined effect of the HBD and the HBA of NADES on the enhancement of thermostability.^20^

The half-life of laccases at 70 °C with or without the 2Bet:1Sor NADES preincubation is reported in Table 2. In general, all the tested laccases benefited from the incubation in the NADES solution, with an increase in their half-life of at least two times compared to the reference solution, as in the cases of EV4 and EV5. Additionally, EV1 and EV3 half-lives was extended three times and those of POXA1b and EV2 nearly four times. In particular, EV5 and EV2 exhibited the longest half-lives at 70 °C when incubated in the NADES solution.

**Table 2:** Half-Lives of Laccases (min) in Reference Solution (50 mM Phosphate Buffer, pH 7) and Aqueous Solution in Presence of 25 wt % of 2Bet:1Sor at 70 °C^a^

	*t*_1/2_ 70 °C in reference solution (min)	*t*_1/2_ 70 °C in 25 wt % 2Bet:1Sor DES (min)
POXA1b	23	89
EV1	22	61
EV2	28	107
EV3	25	76
EV4	22	57
EV5	40	105

a Data refer to five replicates, and standard deviation is less than 5%.

It is worth noting that the thermostabilizing effect of NADES is quite specific for each enzyme variant. Although starting from a similar *t*_1/2_, the achieved increment is different for all the enzymes, pointing out the importance of the specificity of the established interactions between proteins and NADES components.

To get further insights into the observed experimental data, the changes in the Gibbs free energy values (ΔΔ*G*, kcal mol^–1^) due to amino acid substitution in each laccase variants with respect to the wild type POXA1b were calculated using the FoldX method. A beneficial effect of amino acid substitutions was found in the case of the EV5 variant (ΔΔ*G* = −8.35 kcal mol^–1^), while it was almost negligible for the other ones. Thus, the NADES action seems to be effective both in further boosting an already thermostable enzyme (as in the case of EV5) and in compensating for a more thermolabile one (as in the case of EV2).

### Effect of 25 wt % 2Ber:1Sor NADES on Laccases Thermostabilities at 90 °C

**Figure 6:**
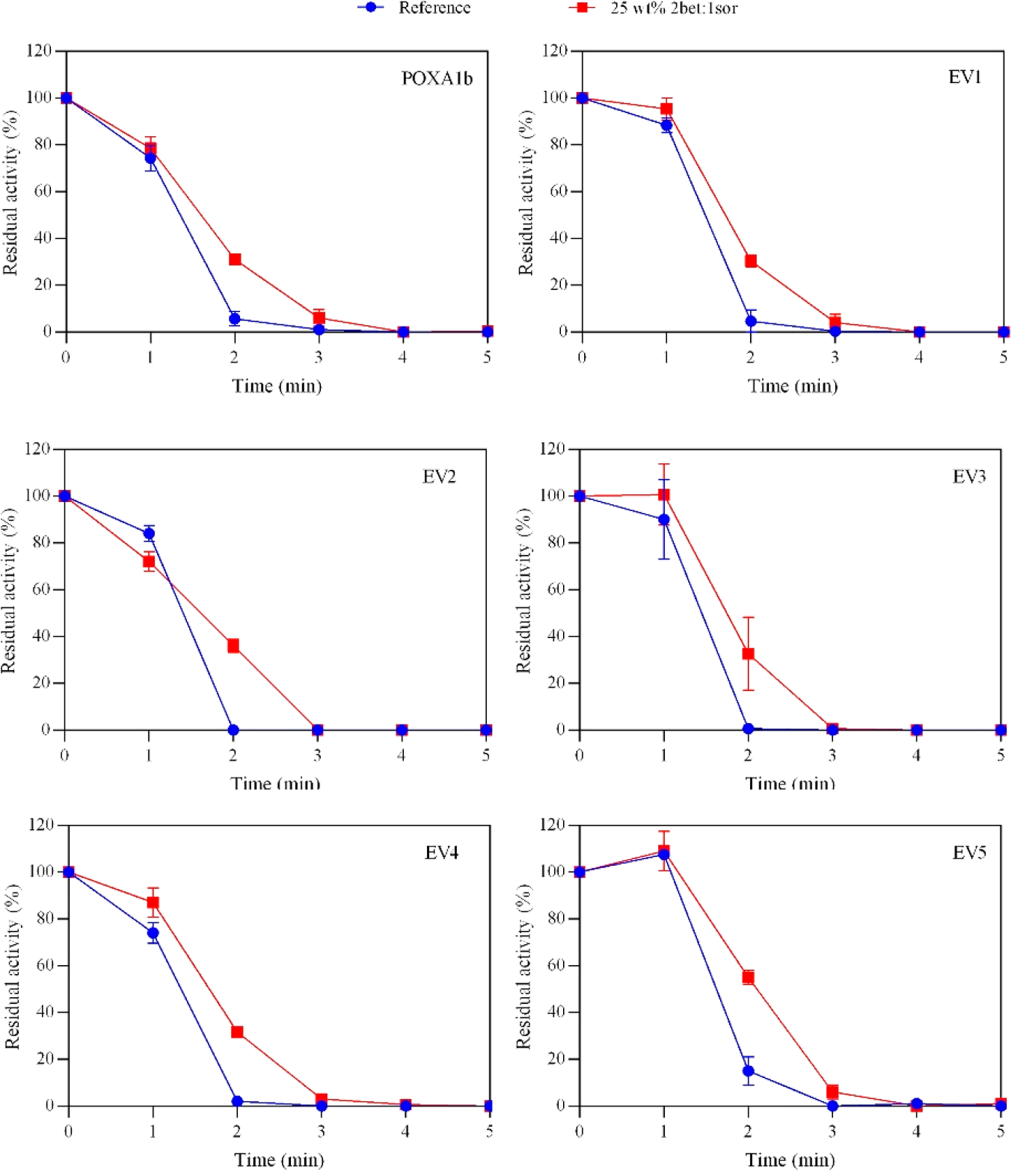
Comparison of thermostability of 1.25 g L^–1^ laccases in 50 mM phosphate buffer, pH 7 aqueous solution in the absence (blue dots), and in the presence of 25 wt % of 2Bet:1Sor (red squares) at 90 °C. The residual activity (%) is determined by comparing the activity measured after a set amount of time with the free-DES phosphate buffer, pH 7.0 and 50 mM at 25 °C (reference, 100%).

The positive effects of 25 wt % 2Bet:1Sor NADES on laccases thermostabilities were also evaluated increasing the temperature of incubation to 90 °C (Figure 6). In general, the incubation in NADES promoted the increase in thermostability of all the tested laccases even at 90 °C. In particular, EV5 showed the highest residual activity with a nearly 45% increase after 3 min incubation in 25 wt % 2Bet:1Sor. These findings could be exploited in applications in which the enzymes are exposed to very high temperature for a few minutes, such as in the extrusion process required to incorporate these enzymes in smart-multilayer plastics.^20^

## Conclusions

POXA1b and five laccase variants were preincubated in five different betaine-based NADESs aqueous media, and their thermal inactivations were monitored at 70 and 90 °C. A clear advantage in the laccase residual activity was observed in the presence of NADES. Supported by the finding that HBD and HBA are single molecules in the liquid phase, individual interactions between single components of NADES and enzymes were evaluated through a molecular docking approach, finding a correlation between the binding energies between NADES and laccase components and the stabilization of the enzymes.

The precise combination of interactions and molecule orientation determined a different stabilizing effect for each enzyme, suggesting the possibility to tailor the NADES composition for every enzyme of interest, taking advantage of computer-aided approaches for a preliminary screening of different combinations of HBD and HBA.

The greenest aspect of the study relies on the possibility to use inherently nonhazardous and renewable solvents, such as NADES, to promote laccase-catalyzed reactions in fields requiring high operating temperatures, such as the biofuel industry^46^ and processing of smart-materials containing enzymes^20^ as well as in the conversion of nonaqueous soluble substrates. In this regard, laccase activity may be easily affected by the solvent system due to the proximity of the active site to the surface of the enzyme.^47^ The combination of NADES and enzymatic catalysis further reinforces the sustainability of the process, since enzyme thermo-protection allows for minimizing the costs associated with enzyme production while ensuring high process efficiency.

We expect such an approach to address the green chemistry principles by promoting an environmentally friendly route for a wide range of reaction processes.

## Supporting Information Available

Laccase activity in five different betaine-based NADES (Figure S1). Thermodynamic cycle of sorbitol-betaine-based NADES (Figure S2). Superposition of POXA1b, 1GYC, 2H5U, 2XYB, 3DIV, and 3FPX laccase structures (Figure S3). Localization of HBA and HBA interactions (Table S1). (PDF)

## AbbreviationS

ABTS: 2,2′-azino-bis(3-ethylbenzothiazoline-6-sulfonic acid)
Bet: betaine
Ery: meso-erythritol
EtG: ethylene glycol
Gly: glycerol
HDA: hydrogen bond acceptor
HBD: hydrogen bond donor
NADES: natural deep eutectic solvents
PMF: potential mean force
Sor: sorbitol
Xyl: xylitol
