# Supplementary material for: Enhancing the Thermostability of Engineered Laccases in Aqueous Betaine-Based Natural Deep Eutectic Solvents
Source: ACS Sustain Chem Eng. 2021 Dec 29;10(1):572–81. doi: 10.1021/acssuschemeng.1c07104 (PMC8753991; doi:10.1021/acssuschemeng.1c07104)
Supplement: Supplementary file 1 — sc1c07104_si_001.pdf [file sc1c07104_si_001.pdf]

Supplementary materials

Enhancing the thermostability of engineered laccases in aqueous betaine-based Natural Deep Eutectic Solvents

Simona Varriale[a], *Astrid E. Delorme*[b], *Jean-Michel Andanson*[b], *Julien Devemy*[b], *Patrice Malfreyt*[b], *Vincent Verney*[b], *Cinzia Pezzella* *[a,c]

[a.]Biopox srl, Viale Maria Bakunin 12, Naples, 80125, Italy.

[b.]Université Clermont Auvergne, CNRS, SIGMA Clermont, ICCF, F-63000 Clermont-Ferrand, France.

[c.]Department of Agricultural Sciences, University of Naples "Federico II", Via Università, 100 Portici, 80055, Italy.

*Corresponding author

Number of pages: 7

Number of figures: 3

Number of tables: 1

Figure S1 Comparison of relative activity of 1.25 g L-1 laccases in 50 mM, pH 7 phosphate buffer aqueous solution in the presence of 25 wt% betaine-based DES obtained using 5 different HBD (sorbitol, xylitol, glycerol, ethylene glycol, erythritol). Relative activity (%) is determined by comparison with the free-DES phosphate buffer pH 7.0 and 50 mM at 25 ˚C (reference, 100%).

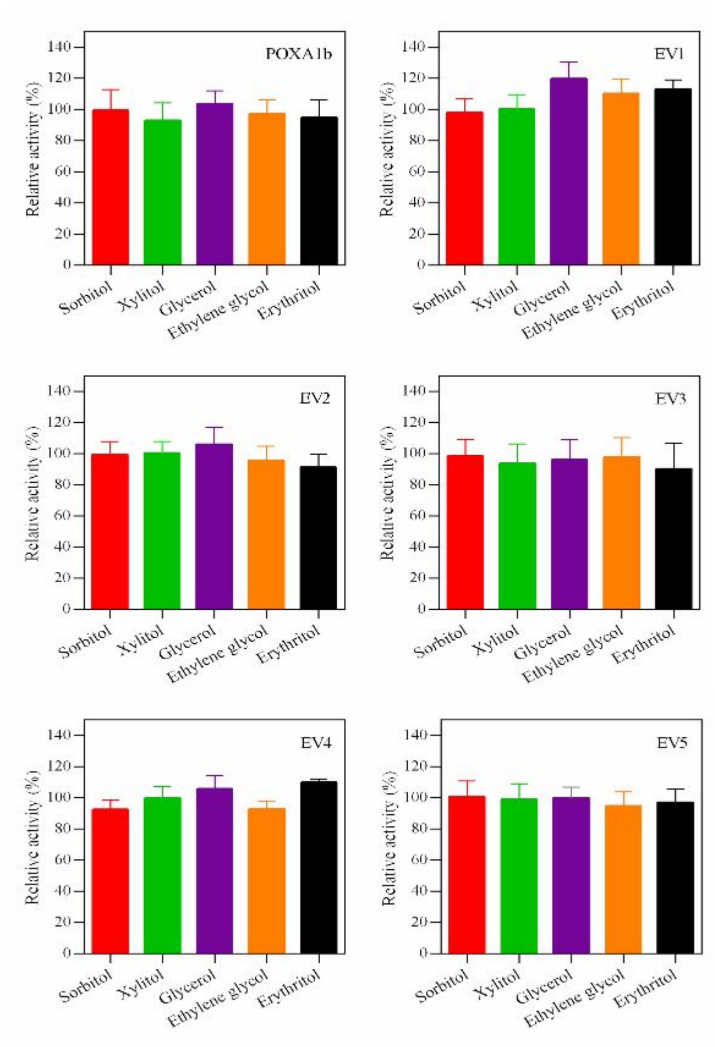

Figure S2 Thermodynamic cycle of sorbitol-betaine based NADES

Thermodynamic cycle:

$$\begin{array}{ccc} \text{Sor (gas) + Bet (gas)} & \xrightarrow{\Delta G_1} & \text{Sor...Bet (gas)} \\ \Delta G_3 \downarrow \quad \Delta G_4 \downarrow & & \uparrow \Delta G_5 \\ \text{Sor (water) + Bet (water)} & \xrightarrow{\Delta G_2} & \text{Sor...Bet (water)} \end{array}$$

$\Delta G_1 - \Delta G_2 = \Delta G_3 + \Delta G_4 + \Delta G_5 = \Delta G^{\circ}_{hyd}$ (Sor) + ΔG°_{hyd} (Bet) - ΔG°_{hyd} (Sor..Bet)

$\Delta G_1 - \Delta G_2 = - 4.7 - 0 < 0$ then

ΔG°_{hyd} (Sor) + ΔG°_{hyd} (Bet) < ΔG°_{hyd} (Sor..Bet)

Figure S3 Superposition of POXA1b, 1GYC, 2H5U, 2XYB, 3DIV, and 3FPX laccase structures. The L1 region of each laccase protein is shown in different colours. Other parts of laccase structures are also shown in different shades of grey.

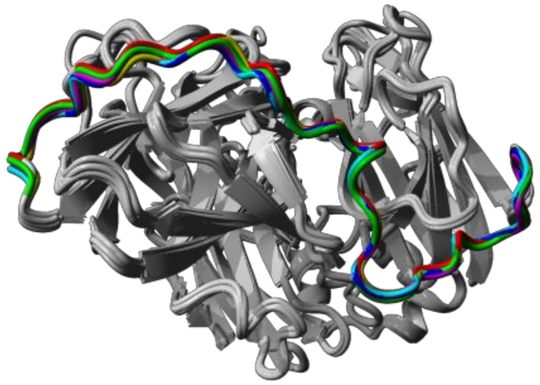

Table S1 Positions of laccases aminoacids engaged in the interactions with HBA and HBDs. Interactions identified using YASARA Structure were hydrogen bonds (blue), hydrophobic interactions (red), cation-π interactions (orange) and ionic interactions (green). The number of interactions is reported in parenthesis.

	POXA1b		EV1		EV2		EV3		EV4		EV5	
	Loop L1	Other	Loop L1	Other	Loop L1	Other	Loop L1	Other	Loop L1	Other	Loop L1	Other
Betaine	Q293(X2)<br>D233(X3)<br>T216<br>G214(X3)			D205<br>W455<br>H162(X4)<br>I453<br>W455(X9)<br>H456		T147<br>P32<br>V145(X2)<br>N171(X2)<br>Y185		Y185<br>N171(X2)<br>V145(X2)<br>P123<br>E142(X2)		T147<br>E142<br>V145<br>Y185<br>N171<br>P32		M499(X2)<br>T501<br>T55<br>V59(X5)<br>N502<br>T501
Sorbitol	Q293(X3)<br>T216<br>S211			A103<br>N226<br>G224<br>N226<br>E225	T216<br>G214<br>D233(X2)<br>Q234<br>A295		D233<br>E292<br>S211<br>G214		G214<br>Q293<br>E292<br>S211		K310<br>A103<br>N226<br>Q102<br>F69	

			S211			T402
Xylitol	Q293<br>D233<br>Q293(X2)<br>S211	Q293<br>Q293(X2)<br>A295<br>E292<br>S211	H215<br>G214<br>S211<br>Q293(X2)<br>D233<br>A295<br>Q234	Q293<br>E292<br>T216<br>D233	D213<br>S211<br>E292<br>Q293(X3)	T402<br>F69<br>G224
Glycerol	D150<br>S206<br>Y208<br>P262(X3)<br>D266	D233<br>H215<br>A295<br>Q294<br>S211<br>Q293(X3)<br>Q234	I212<br>D213<br>Q293(X2)<br>S211<br>E292	D233<br>Q293<br>E292<br>Q293(X2)<br>D233<br>S211	I212(X2)<br>Q293<br>D233<br>Q293(X2)<br>E292	N415<br>P419<br>Y302<br>V405<br>S408

Ethylene Glycol	F238<br>P395(X2)	S411<br>S408<br>N415(X2)<br>S408(X2)<br>Y302(X2)	S408/S408<br>S411<br>N415(X2)<br>Y302	Y137<br>D140<br>K134	E301(X2)<br>L299(X2)<br>F238	P165<br>P262<br>D266<br>G265
Erythritol	E292<br>Q293<br>D233<br>S211	I212<br>Q293(X2)<br>S211<br>Q234<br>A295	H215<br>D213<br>D233/D233<br>S211<br>E292<br>Q234<br>A295<br>D293(X2)	Q293<br>E292<br>Q293<br>D233<br>S211	N294<br>S211<br>Q293<br>E292<br>D233<br>T216	D233/D233<br>G214<br>Q293(X2)<br>S211<br>A295<br>Q234
